# *IPA1* Negatively Regulates Early Rice Seedling Development by Interfering with Starch Metabolism via the GA and *WRKY* Pathways

**DOI:** 10.3390/ijms22126605

**Published:** 2021-06-20

**Authors:** Yonggang He, Menghao Zhu, Zhihui Li, Shan Jiang, Zijun He, Shuang Xu, Xiangsong Chen, Zhongli Hu, Zhihong Zhang

**Affiliations:** State Key Laboratory of Hybrid Rice, College of Life Sciences, Wuhan University, Wuhan 430072, China; whuhyg@whu.edu.cn (Y.H.); 2012202040102@whu.edu.cn (M.Z.); 2020102040041@whu.edu.cn (Z.L.); 2020202040061@whu.edu.cn (S.J.); 18986642398@163.com (Z.H.); 2020202040067@whu.edu.cn (S.X.); chen.xs@whu.edu.cn (X.C.); huzhongli@whu.edu.cn (Z.H.)

**Keywords:** rice (*Oryza sativa* L.), seed germination, *IPA1*/*OsSPL14*, gibberellin (GA), *WRKY*

## Abstract

*Ideal Plant Architecture 1 (IPA1)* encodes SQUAMOSA PROMOTER BINDING PROTEIN-LIKE 14 (SPL14) with a pleiotropic effect on regulating rice development and biotic stress responses. To investigate the role of *IPA1* in early seedling development, we developed a pair of *IPA1*/*ipal*-NILs and found that seed germination and early seedling growth were retarded in the *ipa1*-NIL. Analysis of the soluble sugar content, activity of amylase, and expression of the *α*-amylase genes revealed that the starch metabolism was weakened in the *ipa1*-NIL germinating seeds. Additionally, the content of bioactive gibberellin (GA) was significantly lower than that in the *IPA1*-NIL seeds at 48 h of imbibition. Meanwhile, the expression of GA synthesis-related gene *OsGA20ox1* was downregulated, whereas the expression of GA inactivation-related genes was upregulated in *ipa1*-NIL seeds. In addition, the expression of *OsWRKY51* and *OsWRKY71* was significantly upregulated in *ipa1*-NIL seeds. Using transient dual-luciferase and yeast one-hybrid assays, IPA1 was found to directly activate the expression of *OsWRKY51* and *OsWRKY71*, which would interfere with the binding affinity of GA-induced transcription factor OsGAMYB to inhibit the expression of *α*-amylase genes. In summary, our results suggest that *IPA1* negatively regulates seed germination and early seedling growth by interfering with starch metabolism via the GA and *WRKY* pathways.

## 1. Introduction

Seed germination is a complex and diverse process that plays an important role in ensuring the continuity of life. Generally, germination starts with water uptake and the reactivation of diverse metabolism processes in the quiescent seed, followed by emergence of the radical and coleoptile [1]. In rice, many nutrients accumulate in the endosperm during seed maturation. As germination progresses, these reserves are hydrolyzed into metabolizable nutrients by a series of hydrolases, then absorbed by the scutellum epithelium and transported to the embryonic axis [2,3]. Thus, the efficient degradation and utilization of seed reserves are particularly important to seed germination and early seedling development.

Starch is the major reserve in rice seed that provides a major carbon source for generating energy and metabolites. During seed germination and early seedling growth, starch is degraded by the concerted action of various hydrolases, including *α*-amylase, *β*-amylase, debranching enzyme, and *α*-glucosidase [4]. Among them, *α*-amylase is the most abundant hydrolase in rice. It catalyzes the hydrolysis of *α*-1,4-glucosidic bonds of starch at random sites to generate *α*-glucose and *α*-maltose [5,6]. After seed imbibition, *α*-amylase is synthesized in the aleurone layer and scutellum to break down starch granules. Generally, the expression of rice α-amylase gene *αAmys* (also called *RAmys*) is positively and negatively regulated by gibberellin and sugar, respectively [7,8]. After imbibition, GA induces the expression of *OsGAMYB* (also called *MYBGA*), which directly binds to the GA response element (GARE) and activates the expression of *αAmys* [9,10]. Meanwhile, the expression of *αAmys* is strongly induced by sugar starvation and repressed by various sugars produced during mobilization of endosperm stores [7,11,12]. These results show the complicated regulatory mechanisms of α-amylase.

The GA contents in germinating seeds are determined by GA biosynthesis and metabolism. In brief, GA biosynthesis is catalyzed by the combined actions of ent-copalyl diphosphate synthase (CPS), ent-kaurene synthase (KS), ent-kaurene oxidase (KO), ent-kaurene acid oxidase (KAO), GA 20-oxidase (GA20ox), and GA 3-oxidase (GA3ox), whereas it is inactivated by GA 2-oxidase (GA2ox) [13,14]. The endogenous bioactive GA contents and seed germination rates are correlated with the expression levels of these enzyme-related genes. In a *germination-defactive1* (*gd1*) mutant, the expressions of GA biosynthesis-associated genes *OsGA20ox1*, *OsGA20ox2*, and *OsGA3ox2* were suppressed, while the expression of the GA inactivation gene *OsGA2ox3* was dramatically upregulated, resulting in decreased endogenous GA_4_ content and the inhibition of seed germination [15]. Meanwhile, the GA content was significantly lower in the germinating seed embryo of an *OsGA20ox2* loss-of-function mutant, which resulted in a delayed seed germination [16].

In *Arabidopsis* and rice, changes in the expression level of miR156 affect seed dormancy [17,18]. As a target of miR156, the *Ideal Plant Architecture 1* (*IPA1*), which encodes a transcription factor OsSPL14, a member of the SQUAMOSA PROMOTER BINDING PROTEIN-LIKE (SPL) family in rice, is an important regulator of plant development [19,20,21]. In *ipa1* mutant plants, one nucleotide substitution located in the miR156 recognition site perturbs the miR156-regulated degradation of *IPA1* mRNA, leading to an ideal plant architecture with reduced tiller number, increased lodging resistance and panicle branches and, thus, an increase in yield [19,20]. In addition, *IPA1* was also identified as a vital regulator to participate in biotic stress response [22,23]. Here, with a pair of *IPA1*/*ipa1*-NILs developed in our laboratory, we found that *IPA1* negatively regulates the processes of seed germination and early seedling growth. To elucidate the mechanism, we examined the amylase activity, GA content, and expression of the related genes in the NILs. Meanwhile, the relationship between IPA1 and two transcriptional repressors (WRKY51 and WRKY71) was investigated, respectively. Our work suggests that the elevated levels of *IPA1* expression retards seed germination and early seedling growth through GA and *WRKY* pathways. These findings provide new insight into understanding the function of *IPA1* in seedling development.

## 2. Results

### 2.1. ipa1 Retards Seed Germination and Early Seedling Growth of Rice

**Figure 1:**
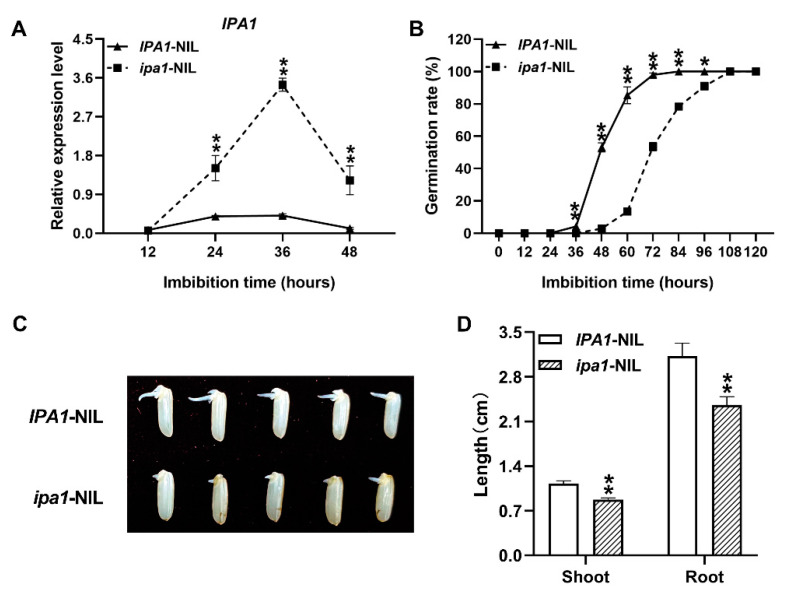
*IPA1* participates in seed germination and early seedling growth in rice. (**A**) The relative expression level of *IPA1* in the NILs. (**B**) Seed germination was comparatively delayed in *ipa1*-NIL plants. (**C**) Morphology of dehulled seeds at 48 h after initial imbibition. (**D**) The shoots and roots were significantly shorter in *ipa1*-NIL plants compared with *IPA1*-NIL plants after 96 h germinated in the Petri dishes at 30 °C. Values are means ±SE (*n* = 3). More than 50 seeds were measured in each replicate. Significant differences were determined using Student’s *t*-test (* *p* < 0.05, ** *p* < 0.01).

To investigate the role of the *IPA1* gene in rice seedling development, we developed a pair of *IPA1*/*ipa1*-NILs and compared the expression pattern of *IPA1* in germinating seeds. We found that *IPA1* expression was significantly higher in the *ipa1*-NIL seeds after 24 h imbibition compared with the *IPA1*-NIL seeds (Figure 1A). In a germination experiment, the initiation time of seed sprouting was delayed by approximately 12 h compared to that of the *IPA1*-NIL seeds (Figure 1B). Meanwhile, the percentage of germinated *ipa1*-NIL seeds was significantly decreased from 36 to 96 h after the initial imbibition (Figure 1B). Additionally, the plateau values of seed germination peaked later for *ipa1*-NIL, although no significant difference was found in germination percentage after 108 h (Figure 1B). Moreover, with the dehulled seeds of NILs, we found that the shoot was significantly decreased in the *ipa1*-NIL seeds at 48 h after initial imbibition (Figure 1C). Then, the root and shoot lengths were recorded for intact seeds. At 96 h after germination in Petri dishes, the shoot length was decreased by 22.66% and the root length was decreased by 24.53% in the *ipa1*-NIL seedlings compared with those of the *IPA1*-NIL seedlings (Figure 1D). Overall, these results indicate that *IPA1* participates in seed germination and early seedling growth in rice and that *ipa1* could retard these processes.

### 2.2. IPA1 Negatively Regulates Starch Metabolism during Seed Germination and Early Seedling Growth

**Figure 2:**
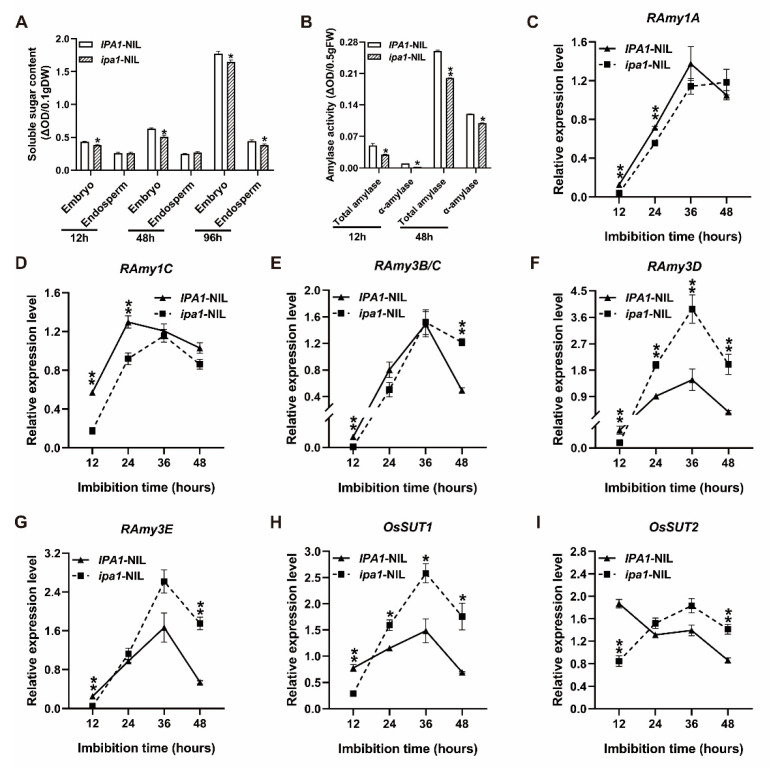
The impacts of *IPA1* on starch metabolism during germination and early seedling growth. (**A**) The soluble sugar content of NILs in the embryo and endosperm at different time points. (**B**) The amylase activity in NILs. (**C**–**G**) The relative expression level of *α*-amylase genes *RAmy1A* (**C**), *RAmy1C* (**D**), *RAmy3B/C* (**E**), *RAmy3D* (**F**), and *RAmy3E* (**G**). (**H**,**I**) The relative expression level of sugar transport-related genes *OsSUT1* (**H**) and *OsSUT2* (**I**). Significant differences were determined using Student’s *t*-test (* *p* < 0.05, ** *p* < 0.01).

Sugar is a direct source of energy for seedling vigor. To investigate whether *IPA1* regulates seed germination and early seedling growth by modulating sugar accumulation, the sugar content was detected in *IPA1*/*ipa1*-NILs. We found that the soluble sugar content of the *ipa1*-NIL seeds was decreased by 11.63%, 19.05%, and 6.80% after 12, 48, and 96 h imbibition, in the embryo, respectively (Figure 2A), while no significant difference between the NILs was found in the endosperm except for 96 h after initial imbibition (Figure 2A). Amylase is a major enzyme involved in the hydrolysis of starch to soluble sugar during seed germination and seedling growth. At 12 and 48 h of imbibition, the total amylase activity in *ipa1*-NIL seeds was decreased by 40% and 23.08%, respectively, with the *α*-amylase activity decreasing by 73.67% and 16.67%, respectively (Figure 2B). Then, the transcript levels of genes encoding α-amylases were measured. The expression of *RAmy1A* and *RAmy1C* was significantly lower in *ipa1*-NIL seeds at 12–24 h of imbibition (Figure 2C,D). Meanwhile, at 12 h of imbibition, *RAmy3B/C*, *RAmy3D*, and *RAmy3E* were significantly downregulated in the *ipa1*-NIL seeds (Figure 2E–G). It has been reported that the *RAmy3* subfamily of genes, especially *RAmy3D*, are induced by sugar starvation during germination and early seedling growth [24,25]. In the present study, the expression of *RAmy3D* was induced quickly in the *ipa1*-NIL seeds, and the levels of *RAmy3D* were 2.16-, 2.63-, and 5.21-fold higher compared to that in the *IPA1*-NIL seeds at 24, 36, and 48 h after imbibition, respectively (Figure 2F). Similarly, *RAmy3B/C* and *RAmy3E* were also upregulated in the *ipa1*-NIL seeds at 48 h after imbibition (Figure 2E,G). Additionally, two sucrose transporter genes *OsSUT1* and *OsSUT4* were found to be downregulated by 62.72% and 54.86% in *ipa1*-NIL, respectively, at 12 h of imbibition, and then they were induced to be upregulated (Figure 2H,I), the same as to that of *RAmy3D*. Taken together, these results suggest that *IPA1* negatively regulates starch metabolism during germination and early seedling growth.

### 2.3. The Retarding Effect of Seed Germination and Early Seeding Growth in the ipa1-NIL Seeds Caused by GA Defect

**Figure 3:**
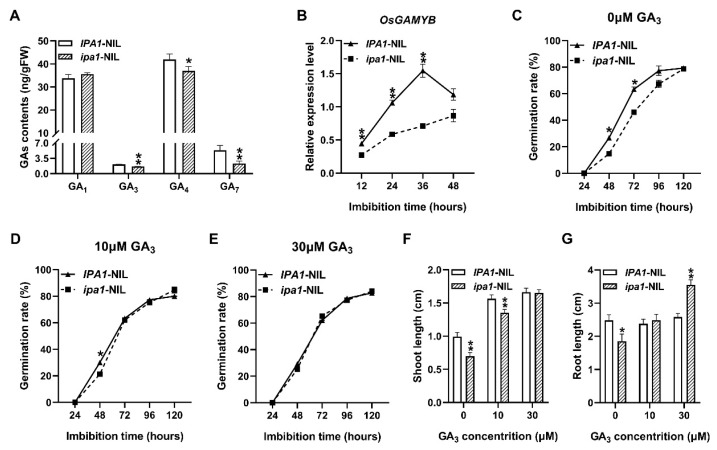
Retarded seed germination and early seedling growth in *ipa1*-NIL seeds is caused by GA defects. (**A**) The contents of bioactive GAs in the seeds after 48 h imbibition. Values are means ±SE (*n* = 3). (**B**) The relative expression level of *OsGAMYB* at different time points. Values are means ±SE (*n* = 3). (**C**–**E**) Germination rate of NILs soaked in 0 μM (**C**), 10 μM (**D**), and 30 μM (**E**) GA_3_ solution. Values are means ±SE (*n* = 3). More than 50 seeds were measured in each replicate. (**F**,**G**) The shoot (**F**) and root (**G**) lengths of seedlings grow on filter paper soaked with water or GA_3_ solution. Values are means ±SE (*n* = 36). Significant differences were determined using Student’s *t*-test (* *p* < 0.05, ** *p* < 0.01).

In germinating grains, bioactive GAs are synthesized in the embryo and transported to the aleurone layer to trigger *αAmy*s expression [26]. In our study, the total contents of the bioactive GAs were markedly decreased in the *ipa1*-NIL seeds at 48 h of imbibition (Figure 3A). Among them, the contents of GA_3_, GA_4_, and GA_7_ were decreased by 22.86%, 11.90%, and 56.18%, respectively (Figure 3A). It has been reported that GAs could induce the expression of *OsGAMYB*, thereby upregulating the expression of the *α*-amylase genes [25]. In the present study, the expression of *OsGAMYB* was downregulated significantly in *ipa1*-NIL seeds during germination (Figure 3B). Meanwhile, when exogenous GA_3_ was applied, the germination rate of the *ipa1*-NIL seeds was markedly accelerated (Figure 3C–E), and the shoot and root lengths of the *ipa1*-NIL seeds were significantly increased after 96 h germinated on filter paper soaked with GA_3_ solution (Figure 3F,G). These data suggest that *ipa1* retards the seed germination and early seedling growth at least via a mechanism mediated by a reduction in the abundance of bioactive GAs.

### 2.4. Enhanced GA Deactivation in the ipa1-NIL

**Figure 4:**
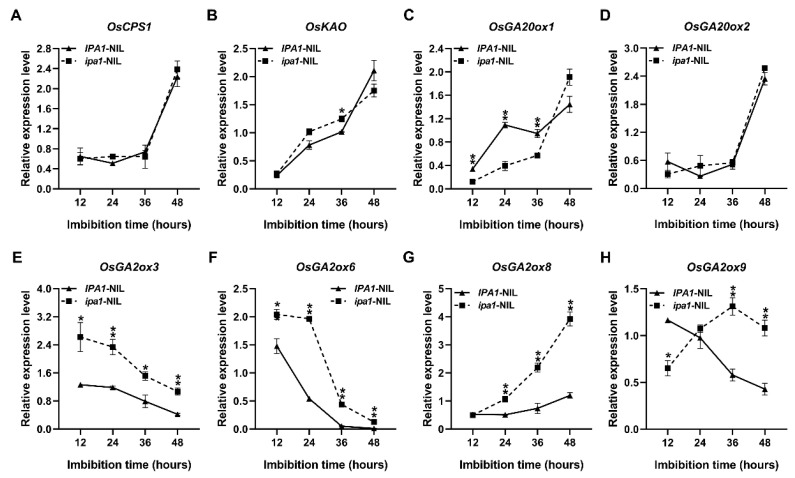
The transcript levels of genes related to GA biosynthesis and inactivation at different time points. (**A**–**D**) The relative expression level of GA biosynthesis-related gene *OsCPS1* (**A**), *OsKAO* (**B**), *OsGA20ox1* (**C**), and *OsGA20ox2* (**D**). (**E**–**H**) The relative expression level of GA metabolism-related gene *OsGA2ox3* (**E**), *OsGA2ox6* (**F**), *OsGA2ox8* (**G**), and *OsGA2ox9* (**H**). Values are means ±SE (*n* = 3). Significant differences were determined using Student’s *t*-test (* *p* < 0.05, ** *p* < 0.01).

Bioactive gibberellins are regulated by changes in biosynthetic and deactivation processes. To investigate how the bioactive GAs were decreased in the *ipa1*-NIL seeds, the expression of GA biosynthetic and inactivated genes was analyzed in the NILs (Figure 4). *OsGA20ox1*, a representative gene in the third step of GA biosynthesis, was significantly downregulated in the *ipa1*-NIL during 12–36 h of seed imbibition (Figure 4C), although no significant difference was observed in the expression levels of the GA synthesis-related genes *OsCPS1*, *OsKAO* and *OsGA20ox2* between the NILs except for *OsKAO* at the point of 36 h (Figure 4A,B,D). The GA2ox enzymes are involved in GA deactivation in rice. In this study, the *OsGA2ox3*, *OsGA2ox6*, and *OsGA2ox8* were upregulated in *ipa1*-NIL during almost the entire imbibition period (Figure 4E,G), and *OsGA2ox9* also showed a higher expression level in *ipa1*-NIL after 36 h, although it was downregulated in the beginning (Figure 4H). Overall, elevated expression of *IPA1* enhanced the deactivation process of GAs during seed germination and early seedling growth.

### 2.5. IPA1 Directly Binds to OsWRKY51 and OsWRKY71 and Promotes Their Expression

**Figure 5:**
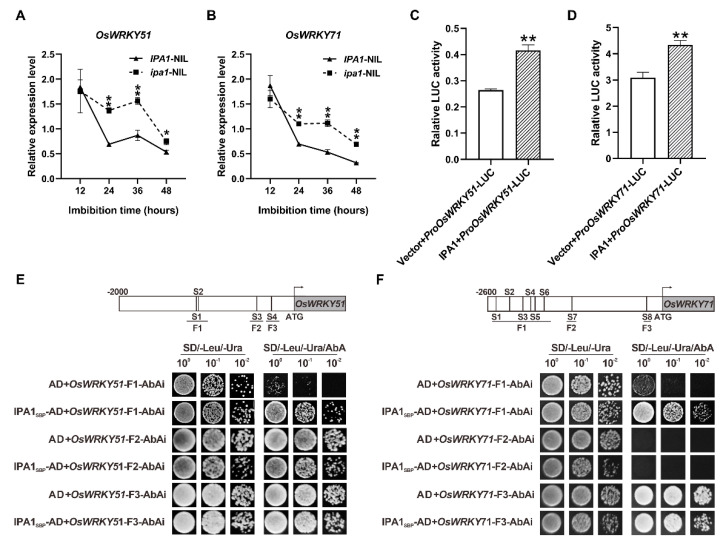
IPA1 positively regulates the expression of *OsWRKY51* and *OsWRKY71*. (**A**,**B**) The relative expression level of *OsWRKY51* (**A**) and *OsWRKY71* (**B**) at different time points. Values are means ±SE (*n* = 3). (**C**,**D**) Relative LUC activities of *ProOsWRKY51* (**C**) and *ProOsWRKY71* (**D**) reporter after co-expression with *IPA1* in rice protoplasts, respectively. The pRGV vector was used as control. Relative LUC activity was calculated by LUC/Ren. Values are means ±SD (*n* = 3). Significant differences were determined using Student’s *t*-test (* *p* < 0.05, ** *p* < 0.01). (**E**,**F**) Y1H assays to dissect the binding regions of IPA1 in the promoter regions of *OsWRKY51* (**E**) and *OsWRKY71* (**F**), respectively. The core GTAC motifs are marked as S1, S2, and so on.

Previous studies have demonstrated that OsWRKY51 and OsWRKY71 could suppress the GA-induced expression of *α*-amylase genes by interfering with the transactivator OsGAMYB [27], and IPA1 was found to be enriched in the *OsWRKY51* promoter [28]. In this study, we found that the expression levels of *OsWRKY51* and *OsWRKY71* in *ipa1*-NIL seeds were significantly upregulated after 24 h imbibition (Figure 5A,B). By transient dual-luciferase (LUC) assays with rice protoplasts, we found that IPA1 promoted expression of the luciferase reporter gene driven by the *OsWRKY51* and *OsWRKY71* promoter, respectively (Figure 5C,D). As a transcription factor, IPA1 could regulate the expression of its target genes by binding to the core motif GTAC and/or TGGGCC/T in promoters [28]. Then, we conducted sequence analyses and identified four and eight GTAC motifs in the *OsWRKY51* and *OsWRKY71* promoter, respectively (Figure 5E,F). Yeast one-hybrid (Y1H) assays were performed to evaluate if IPA1 directly binds to the promoters of *OsWRKY51* and *OsWRKY71*. The cells co-transformed with bait and the prey vectors could grew well on SD/−Leu/−Ura/AbAi plates (Figure 5E,F), indicating that IPA1 can directly bind to the promoters of *OsWRKY51* and *OsWRKY71*. Taking these results together, we conclude that *OsWRKY51* and *OsWRKY71* are the direct target genes of IPA1.

## 3. Discussion

*IPA1*/*OsSPL14* is a pleiotropic gene that plays an intricate role in rice plant development and stress response [19,23,29]. In *ipa1* plants, a point mutation in the miR156 recognition site perturbed the miR156-regulated degradation of *IPA1* mRNA, leading to changed plant architecture [19]. Previous studies have reported that the overexpression of *IPA1* or knockout of miR156 enhances seed dormancy [18,22]. Here, we found that the expression level of *IPA1* was rapidly upregulated in the soaked *ipa1*-NIL seeds (Figure 1A). Based on the seed germination rate as well as shoot and root lengths of NILs (Figure 1B–D), our study suggested that the higher expression level of *IPA1* in *ipa1*-NIL delayed seed germination and retarded early seedling growth.

Seed germination and embryo growth rely on the energy supplied by starch decomposition. In the endosperm, starch is decomposed into soluble sugars that are transported into the germinating embryo axis [11,30]. In addition, the enzyme α-amylase is responsible for the degradation of reserve carbohydrate to soluble sugars [31]. In the embryo, soluble sugars provide substrates and energy for fueling embryonic development. In the present study, a lower soluble sugar content was found in the *ipa1*-NIL embryo (Figure 2A). Further analyses found that the activity of α-amylase was significantly decreased in *ipa1*-NIL seeds (Figure 2B). Therefore, the delayed germination and retarded early seedling growth in *ipa1*-NIL seeds could be caused by the suppression of starch metabolism.

It has been reported that GA plays a critical role in promoting seed germination [1]. In plants, GA_1_, GA_3_, GA_4_, and GA_7_ are the major bioactive GA forms [13]. Our results revealed that the contents of GA_3_, GA_4_, and GA_7_ were dramatically decreased in *ipa1*-NIL compared with those in the *IPA1*-NIL seeds (Figure 3A). These findings are consistent with a previous study which showed that the content of GA_3_ and GA_7_ was decreased in the fresh seeds of the miR156 mutant [18]. Therefore, we further investigated the effect of exogenous GA on the seed germination of the NILs and, as a result, GA_3_-promoted germination and seedling growth were clearly observed in *ipa1*-NIL seeds (Figure 3C–G), although previous studies have reported that the exogenous GA application has little effect on the germination of the miR156 mutant or *IPA1*-OE seeds [18,22]. Altogether, these results support the hypothesis that the elevated expression of *IPA1* delays seed germination and retards early seedling growth mainly through the GA pathway.

The contents of bioactive GAs were cooperatively regulated by biosynthesis and inactivation. In rice, *OsGA20ox* and *OsGA2ox* are involved in GA biosynthesis and inactive during seed germination, respectively [15,32]. To explore the mechanisms of how *ipa1* downregulates the content of GAs, the expression levels of GA synthesis- and inactivation-related genes were investigated. For the GA synthesis-related genes, our results demonstrated that *OsGA20ox1* was markedly downregulated in the *ipa1*-NIL seeds during germination (Figure 4C). Although the ChIP-qPCR and EMSA assays demonstrated that IPA1 could directly bind to the GTAC-containing regions in the promoters of the two GA synthesis-related genes *OsCPS1* and *OsKAO* [18], no obvious difference was found in the expression of *OsCPS1* and *OsKAO* between the NILs (Figure 4A,B). For the GA inactivation-related genes, *OsGA2ox3*, *OsGA2ox6*, *OsGA2ox8*, and *OsGA2ox9* were generally upregulated in *ipa1*-NIL. Therefore, the lower expression level of the GA synthesis-related gene *OsGA20ox1* and the higher level of the GA inactivation-related genes could lead to the lower contents of GAs in *ipa1*-NILs seeds.

The *OsGAMYB* gene is a GA-regulated transcription factor required for the transcriptional activation of *α*-amylase genes [7,33]. In the *ipa1*-NIL seeds, the expression level of *OsGAMYB* was significantly lower (Figure 3B). Meanwhile, *RAmy1A* and *RAmy1C* were significantly downregulated during the early stage of germination and *RAmy3B/C*, *RAmy3D*, and *RAmy3E* were also downregulated in the beginning. Interestingly, *RAmy3B/C*, *RAmy3D*, and *RAmy3E*, especially *RAmy3D*, were then upregulated quickly, which might be induced by sugar starvation as reported in the previous studies [24,25]. These observations suggested that *IPA1* negatively regulates the early seedling development of rice by interfering with starch metabolism via the GA pathway.

The GA response in the aleurone cells is influenced by various factors. In recent decades, many members of the WRKY family have been reported to participate in GA-mediated seed germination. In rice, OsWRKY24 can bind to the *Amy32b* promoter and independently suppress GA induction of the *Amy32b* expression [34]. Recently, *OsWRKY72* was identified as a negative regulator in rice germination to suppress GA accumulation through the “*LRK1*-*OsKO2*” pathway in the aleurone layer [35]. OsWRKY51 and OsWRKY71 function as heterologous dimers and directly interfere with GA-induced transcription factor OsGAMYB to inhibit the expression of *αAmys* [27,36]. Although OsWRKY51 does not bind to the *Amy32b* promoter in vitro, it could interact with OsWRKY71 to enhance the binding affinity of OsWRKY71 to the W-box [27]. In the present study, we found that the transcript levels of *OsWRKY51* and *OsWRKY71* were significantly upregulated in *ipa1*-NIL seeds (Figure 5A,B). As a transcription factor, IPA1 could regulate the expression of its target genes by binding to the core motif GTAC and/or TGGGCC/T in promoters [28]. Based on the results of the Y1H and transient dual-luciferase assays, our results suggested that IPA1 can directly activate the expression of *OsWRKY51* and *OsWRKY71* (Figure 5E,F), which further influences the binding affinity of OsGAMYB to the promoter of *αAmys*, thus downregulating its expression.

Recently, the expanding knowledge on *IPA1* has led to the proposal of *IPA1* as a pleiotropic gene to improve agronomic traits in rice [22,23]. Seed germination and early seedling development are the traits we were concerned with here. Generally, when the seeds are soaked in water, their GA level increases dramatically so as to promote seed germination [26]. In our study, the expression of *IPA1* was upregulated quickly in the germinating seeds of the *ipa1*-NIL. Meanwhile, the GA synthesis-related gene *OsGA20ox1* and the GA inactivation-related genes such as *OsGA2ox3*, *OsGA2ox6*, *OsGA2ox8*, and *OsGA2ox9* were detected to be downregulated and upregulated, respectively. As a result, the GA level was decreased and then the expression of *OsGAMYB* was downregulated in *ipa1*-NIL. Additionally, *IPA1* could promote the expression of *OsWRKY51* and *OsWRKY71* directly, which would influence the binding affinity of OsGAMYB to the *α*-amylase genes. Therefore, our results suggested that *IPA1* retards seed germination and early seedling growth by interfering with starch metabolism via both the GA and *WRKY* pathways.

## 4. Materials and Methods

### 4.1. Plant Materials

To generate near-isogenic lines (NILs) of *ipa1* and *IPA1*, the *ipa1-*containing *japonica* line Shaoniejing (SNJ) [19] was crossed with an *indica* cultivar Yuetai B (*IPA1*/*IPA1*) to develop the Yuetai B/SNJ F_1_ plants. Then, the F_1_ plants were backcrossed with Yuetai B to develop the BC_1_F_1_ plants. Subsequently, one of the BC_1_F_1_ plants was self-crossed for six generations to develop Yuetai B/SNJ BC_1_F_7_ plants. Using marker-assisted selection, a BC_1_F_7_ plant with the genotype *IPA1*/*ipa1* on the *IPA1* locus was obtained. In the self-crossed progeny of this BC_1_F_7_ plant, the plants with the genotype *IPA1*/*IPA1* were identified as *IPA1*-NIL, whereas the plants with the genotype *ipa1*/*ipa1* were identified as *ipa1*-NIL.

### 4.2. Seed Germination Analysis

Sterilized NIL seeds were incubated at 30 °C in the dark with water, 10 μM GA_3_, or 30 μM GA_3_. Three replicates (more than 50 seeds/replicate) were analyzed for each treatment. The seed germination rate was calculated at different time points after imbibition. A seed was considered to have germinated when the radicle or shoot length was longer than 1 mm.

### 4.3. Determination of α-Amylase Activity

The amylase activity was quantitatively determined by a modified version of the 3,5-dinitrosalicylic acid method, as described in previous studies [37,38]. Briefly, the seeds were incubated in the water at 28 °C. After 12 and 48 h incubation, the seeds were collected and ground in liquid nitrogen. Powder (0.5 g) was used for enzyme extraction. To determine the *α*-amylase activity, 1 mL crude enzyme extract was incubated in the water at 70 °C for 15 min to inhibit the activity of *β*-amylase. The additional 1 mL enzyme extract was not heated and used to measure the total amylase activity. Then, each enzyme extract was mixed with 1 mL of 1% (*w/v*) soluble starch dissolved in citric acid buffer (pH = 5.6). After heating in a water bath at 40 °C for 5 min, 2 mL 3,5-dinitrosalicylic acid reagent was added, and the mixture was boiled for 5 min. The absorbance of the mixture was measured at wavelengths of 540 nm. Three replicates were analyzed for each treatment.

### 4.4. Measurement of Soluble Sugar Content

The soluble sugar content was quantified by the anthrone method [39] with minor modifications. The embryos and endosperms of germinating seeds were collected from each replicate at 12, 48, and 96 h and immediately dried to a constant weight at 70 °C. Subsequently, the sample was ground, and 0.01 g powder was mixed with 800 μL of 80% (*v/v*) ethanol. The mixture was heated at 80 °C for 30 min, cooled, centrifuged at 3000 rpm (664× *g*) for 10 min, and diluted to a volume of 2 mL with 80% (*v/v*) ethanol. The reaction mix contained 200 μL extract and 1 mL reagent was heated at 95 °C for 15 min. The absorbance of samples was finally recorded at 620 nm. The soluble content was calculated using glucose as the standard. Three replicates were analyzed for each treatment.

### 4.5. Measurement of Endogenous Bioactive GA Content

The endogenous GA contents in seeds were determined as described in [40]. In brief, the dehulled seeds were ground in liquid nitrogen, and 120 mg powder was mixed with 1.2 mL 80% (*v/v*) methanol at 4 °C. After centrifugation at 12,000× *g* for 15 min at 4 °C, the supernatant was dried under a stream of N_2_. Then, the residues were dissolved in 30% methanol and centrifuged. The supernatant was collected for LC-MS analysis.

### 4.6. RNA Extraction and qPCR

After 12, 24, 36, and 48 h incubation, total RNA from the germinating seeds was extracted with TRIzol reagent (Invitrogen, Carlsbad, CA, USA). First-strand cDNA was synthesized from 2 μg RNA in a total of 20 μL reaction system with an ABScript III RT Master Mix with gDNA Remover (ABclonal, Wuhan, China) according to the manufacturer’s instructions. Synthesized cDNAs were used for qPCR with 2× Universal SYBR Green Fast qPCR Mix (ABclonal, Wuhan, China) on a CFX384^TM^ real-time PCR Detection System (Bio-Rad, Hercules, CA, USA). The primers used for qPCR are listed in Table S1. PCR thermal cycling conditions were as follows: initial denaturation, 95 °C for 5 min; 40 cycles of denaturation 95 °C for 10 sec, annealing and extension at 60 °C for 30 s; followed by melting and plate reading. Three biological replicates were included for each sample. The *OsActin* gene expression was used as an internal control to normalize the expression of target genes.

### 4.7. Dual-Luciferase Assay

The dual-luciferase assays were performed according to a previously described method [29]. The promoter of *OsWRKY51* and *OsWRKY71* was amplified from the genomic DNA (primer pairs listed in Table S1) and cloned into the upstream of the LUC reporter gene to generate the *ProOsWRKY51*-LUC and *ProOsWRKY71*-LUC reporter construct, respectively. The luciferase gene from *Renilla reniformis* (Ren) under control of the CaMV 35S promoter was used as an internal control. The cDNA of *IPA1* was amplified (primer pairs listed in Table S1) and inserted into the HindIII/BamHI digested pRGV vector [41] to generate the *Ubi*:*IPA1* effector construct. The combined reporter and effector plasmids were co-transformed into rice protoplasts as described in [42]. An empty pRGV vector co-transformed with the reporter construct was used as a vector control. The LUC activity was measured with the Dual-Luciferase Assay Kit (Promega, Beijing, China) according to the manufacturer’s recommendations, and the relative LUC activity was calculated as the ratio of LUC/Ren.

### 4.8. Yeast One-Hybrid Assay

The coding region of IPA1-SBP was amplified by PCR and cloned into a pGADT7 vector to produce an IPA1_SBP_-AD construct. Various truncated fragments of the promoter regions of *OsWRKY51* and *OsWRKY71* were amplified and ligated into the pABAi vector to generate the bait vectors. After linearization by the *Bst*BI enzyme, the bait vector co-transformed with the prey vector into the yeast strain Y1HGold. The transformants were grown on SD/−Ura/−Leu plates at 30 °C for 3 days, then grown on SD/−Ura/−Leu/AbAi plates. Yeast strains containing the empty pGADT7 in combination with the bait vector were used as the negative control.

## Data Availability

Data are available on request to the corresponding author.

## Supplementary Materials

Supplementary materials can be found at https://www.mdpi.com/article/10.3390/ijms22126605/s1.
